# Identification of Phylogenetic Position in the Chlamydiaceae Family for Chlamydia Strains Released from Monkeys and Humans with Chlamydial Pathology

**DOI:** 10.1155/2010/130760

**Published:** 2010-06-30

**Authors:** Alexander Karaulov, Vladimir Aleshkin, Vladimir Slobodenyuk, Olga Grechishnikova, Stanislav Afanasyev, Boris Lapin, Eteri Dzhikidze, Yuriy Nesvizhsky, Irina Evsegneeva, Elena Voropayeva, Maxim Afanasyev, Andrei Aleshkin, Valeria Metelskaya, Ekaterina Yegorova, Alexandra Bayrakova

**Affiliations:** ^1^Sechenov Moscow Medical Academy, Trubetskaya 8, 119991 Moscow, Russia; ^2^Garbichevsky Moscow G.N. Research Institute of Epidemiology and Micro-Biology, Admiral Makarov 10, 125212 Moscow, Russia; ^3^Research Institute of Medical Primatology, Veseloye-1, 354376 Sochi-Adler, Russia

## Abstract

Based on the results of the comparative analysis concerning relatedness and evolutional difference of the 16S–23S nucleotide sequences of the middle ribosomal cluster and 23S rRNA I domain, and based on identification of phylogenetic position for Chlamydophila pneumoniae and Chlamydia trichomatis strains released from monkeys, relatedness of the above stated isolates with similar strains released from humans and with strains having nucleotide sequences presented in the GenBank electronic database has been detected for the first time ever. Position of these isolates in the Chlamydiaceae family phylogenetic tree has been identified. The evolutional position of the investigated original Chlamydia and Chlamydophila strains close to analogous strains from the Gen-Bank electronic database has been demonstrated. Differences in the 16S–23S nucleotide sequence of the middle ribosomal cluster and 23S rRNA I domain of plasmid and nonplasmid Chlamydia trachomatis strains released from humans and monkeys relative to different genotype groups (group B-B, Ba, D, Da, E, L1, L2, L2a; intermediate group-F, G, Ga) have been revealed for the first time ever. Abnormality in *incA* chromosomal gene expression resulting in Chlamydia life development cycle disorder, and decrease of Chlamydia virulence can be related to probable changes in the nucleotide sequence of the gene under consideration

## 1. Introduction

Chlamydia is obligate intracellular bacteria that replicate only in the cytoplasmic inclusions of the eukaryotic host cells. They are grouped in the Chlamydiales order, Chlamydieceae family; they comprise two kinds, Chlamydia and Chlamydophila, which in turn consist of nine species subdivided into groups depending on whether they cause pathological conditions in human beings. The group of pathogens that infect humans includes the most commonly diagnosed species Chlamydia trachomatis and the least frequent Chlamydophila pneumoniae (formerly Chlamydia pheumoniae); the very rare species Chlamydophila psittaci (formerly Chlamydia psittaci) causes disease in birds. Chlamydophila abortus (formerly Chlamydia abortus) causes miscarriages while Chlamydophila felis causes pneumonia in cats. The species that have never been found to cause medical conditions in humans include: Chlamydophila caviae (formerly Chlamydia caviae) that causes conjunctivitis in guinea pigs, Chlamydophila pecorum (formerly Chlamydia pecorum), Chlamydia suis (formerly Chlamydia trachomatis) that causes disease in pigs, Chlamydophila pecorum (formerly Chlamydia pecorum), and Chlamydia suis (formerly Chlamydia trachomatis), which causes disease in mice [1–3].

In the past the following criteria were used to differentiate between species: the morphology of inclusions, sensitivity to sulfadiazine, ability to synthesise and accumulate glycogen in chlamydial inclusions, and estimates of the DNA-DNA homology. Thus, Chlamydia trachomatis that belongs to the Chlamydia genus and Chlamydophila psittaci that belongs to the Chlamydophila genus were the first two species to be differentiated on the basis of differences in the morphology of inclusions, glycogen synthesis, and sensitivity to sulfadiazine. All chlamydia-like bacteria were classified as either Chlamydia trachomatis or Chlamydia psittaci (Chlamydophila psittaci) of the Chlamydiales order depending on their morphology and development cycle. Strains of Chlamydia trachomatis were identified on the basis of their ability to accumulate glycogen in inclusions and their sensitivity to sulfadiazine; strains of Chlamydia psittaci were those that lacked the ability to accumulate glycogen and could not resist sulfadiazine [1, 4]. The Chlamydophila pneumoniae species was identified as Chlamydophila psittaci because it had similar phenotypic characteristics such as the density of inclusions, inability to synthesise glycogen, and resistance to sulfadiazine. However, later on Chlamydophila pneumoniae was recognised as a separate species as it exhibited ultrastructural differences in the morphology of elementary bodies and DNA-DNA homology compared to other chlamydia [5]. The fourth species to be recognised as a species different from Chlamydophila psittaci (Chlamydia psittaci) in its phenotypic characteristics was Chlamydophila pecorum [2].

The modern classification of bacterial species has been revised and at the moment it is based on genetic methods of estimating the DNA-DNA and rRNA-DNA homologies and comparing the results of sequencing the 16S and 23S sections of rRNA, using the multipoint mapping sequencing method [1, 3, 5–13].

Of all the molecular analysis methods available, comparative sequencing of rRNA or ribosomal DNA is most suitable for studying the phylogeny of chlamydia, which are microorganisms with similar phenotypes. When studying the phylogeny of chlamydia we primarily analyse the sequences 16S–23S in rRNA and 23S rRNA genes. All species of chlamydia are classified as members of the Chlamydiaceae family if the homology of the 16S rRNA gene is more than 90% [12, 14]. Other groups of chlamydia-like organisms exhibit a homology of the 16S rRNA gene with chlamydia of more than 80%. These include strain Simkania Z [8], strain Hall's coccus [7], and strain Candidatus, that is a close relative of Parachlamydia acanthamoebae [6], which were all obtained from amoebas and were previously interpreted as rickettsia. These bacteria were classified as members of the Chlamydiales order because they are obligate intracellular microorganisms with a development cycle similar to that of chlamydia. Studying the homology of the 16S rRNA gene in chlamydia-like organisms may result in the discovery of new groups of organisms, or new strains of chlamydia, which will be classified as Chlamydiales. There are many very known strains of Chlamydophila psittaci, Chlamydia trachomatis, and Chlamydophila pneumoniae obtained from birds, animals, and humans, the sequencing of whose rRNA is described in sundry papers and books [1, 4, 12, 15]. However, nobody has ever had sequences and carried out a phylogenetic analysis of the strains Chlamydia trachomatis and Chlamydophila pneumoniae obtained from monkeys, which would have determined if they were related to the human strains of chlamydia.

The purpose of this study is to carry out a phylogenetic analysis of the 16S–23S midribosome section and the I 23S domain of rRNA in the strains of chlamydia obtained from monkeys and humans suffering from pathologies caused by chlamydia.

## 2. Materials and Methods

We studied two strains of chlamydia obtained from the cervical canal of monkeys. One of the strains did not have plasmids (Chlamydia trachomatis-NPL) and the other had them (Chlamydia trachomatis-PL); this latter strain was obtained from the lungs of a monkey that died from pneumonia (Chlamydophila pneumoniae-PN); the plasmid carrying strain Chlamydia trachomatis-PL2 obtained from the cervical canal of a human being and the strain Chlamydophila pneumoniae-PN2 obtained from the mouth of a human being [16, 17]. The reference strains Chlamydophila pneumoniae-B and Chlamydia trachomatis-Burkhan (Chlamydia trachomatis-PL3) were provided by the State Collection of Chlamydia of the D.I. Ivanovskiy State Virology Research Institute of the Russian Academy of Medical Sciences. 

The midribosome section of the 16S–23S rRNA genes (include domain I) was amplified and sequenced using purpose-built oligonulceotides: forward primer: 16SF2 5′-CCG CCC GTC ACA TCA TGG-3′, forward primer: IGSIGF 5′-ATA ATA ATA GAC GTT TAA GA-3′, and reverse primer: 23R 5′-TAC TAA GAT GTT TCA GTT C-3′ [4].

Amplification was carried out in a 40 mcl mixture comprising: PCR Buffer (×10) : 700 mM Tris-HCl, pH 8.6/25°C, 166 mM (NH_4_)_2_SO_4,_ 25 mM MgCl_2, _0.2 mM dNTPs, and 2.5 U Taq-polymerase, using a GeneAmp 2700 amplifier (Applied Biosystems, US). The size of the products of amplification with primers 16SF2 and 23R, IGSIGF and 23R was 602 and 276 pairs of nucleotides, respectively.

The section with the primers 16SF2 and 23R was amplified under the following conditions: 95°C for3 minutes, then 40 cycles: 94°C for20 seconds, 55°C for 20 seconds., 72°C for 20 seconds, and 72°C for 3 minutes and finally cooling to 6°C with subsequent storage at 10°C. v IGSIGF and 23R was amplified under the following conditions: 95°C for 3 minutes, then 40 cycles: 94°C for 15 seconds, 50°C for 15 seconds, 72°C for 15 seconds, and finally 72°C for 2 minutes followed by cooling to 6°C with subsequent storage at 10°C. 

Following the amplification 6 mcl of the sample was mixed with a 6-unit buffer for loading and then loaded into a 1.5% agarous gel (0.5 mkg/ml EBR). Electrophoresis was conducted at 50 A, 100 V per 1 cm^2^ for 30 minutes. The amplification products were visualised on an ECX-20L transilluminator (Vilber Lourmat, Germany), using a Gel Imager electrophoresis result registration system (Helicon, Russia). 

The nucleotide sequence of the fragments was determined using an ABI Prism 3100 Genetic Analyzer (Applied Biosystems, US) and a BigDye Terminator v3.1 Cycle Sequencing Kit set of sequencing reagents (Applied Biosystems, US), as per the manufacturer's instructions based on PYNNY CJSC (Post genome and nanotechnology innovations, based on the Innovation Centre for Medical Nanobio-technologies of the State Research Institute for Physical and Chemical Medicine of the Ministry of Health of the Russian Federation, Moscow). 

Sequencing was done using both forward primers (16SF2 5′-CCG CCC GTC ACA TCA TGG-3′, IGSIGF 5′-ATA ATA ATA GAC GTT TAA GA-3′), and reverse primers (23R 5′-TAC TAA GAT GTT TCA GTT C-3′), to get more specific results. The results of the sequencing of fragments were analysed and compared with reverse primer sequencing using the Vector NTI Advance 9.0 (PC) software package (http://www.invitrogen.com/site/us/en/home.html).

The nucleotide sequences 16S–23S of the rRNA genes obtained in the experiment were analysed using MEGA (Molecular Evolutionary Genetics Analysis) version 4.1 (http://www.megasoftware.net/) [18]. Multiple straightening of the nucleotide sequences of the analysed strains and isolated sections of RNA was done with other nucleotide sequences of the RNA of different species of chlamydia available in the GenBank NCBI database. Genetic relation was analysed and phylogenetic trees were constructed using the MEGA 4.1 programme employing the Neighbour-Joining method, based on the *p*-distance model doing a Bootstrap Test of Phylogeny (1000 repetitions) and the Maximum Parsimony method. The degree of homology in the 16S–23S sequences of the midribosome section and domain I of the 23S rRNA gene of the studied strains with different species of chlamydia published in GenBank NCBI were analysed using BLAST (http://blast.ncbi.nlm.nih.gov/Blast.cgi/). 

Phylogenetic analysis of the 16S–23S midribosome section and domain I of the 23S rRNA gene was carried out and tree diagrams were built in comparison with representatives of the Chlamydiales order found in GenBank NCBI (http://www.ncbi.nlm.nih.gov/Genbank/): Chlamydophila abortus EBA (U76710), Chlamydophila psittaci 6BC (U68447), Chlamydophila psittaci NJ1 (U68419), Chlamydophila caviae GPIC (D85708), Chlamydophila felis FP Baker (U68457), Chlamydophila pneumoniae N16 (U68426), Chlamydophila pneumoniae TW-183 (U76711), Chlamydophila pecorum E58 (U68433), Chlamydophila pecorum IPA (U68434), Chlamydia trachomatis A/Har-13 (U68438), Chlamydia trachomatis B/TW-5/OT (68440), Chlamydia trachomatis D/UW-3/CX (U68441), Chlamydia trachomatis L2/434/BU (U68443), Chlamydia suis R22 (U68420), Chlamydia suis S45 (U73110), Chlamydia muridarum MoPn (U68436), and Chlamydia muridarum SFPD (U68437). Of these chlamydia strains the following are well-documented plasmid carriers: Chlamydophila psittaci 6BC—pCpA1 [19, 20], Chlamydophila pneumoniae N16—pCpnE1 [19, 20], Chlamydia trachomatis B/TW-5/OT—pCTT1 [21], Chlamydia trachomatis D/UW-3/CX-pCHL1 [22], Chlamydophila felis—pCfe1 [23], Chlamydophila caviae GPIC-pCpGP1 [24], Chlamydia trachomatis A/Har-13-pCTA [25], Chlamydia trachomatis L2/434/BU-pL2 [26], Chlamydia muridarum MoPn-pMoPn [27]. Information about sequenced plasmids is also found in GenBank NCBI. MEGA 4.1 software packaged was used to estimate the evolutionary difference between the sequences and the standard estimation error(s) for the c I of chlamydia. Variation statistics methods based on calculating the arithmetic mean and standard error (*M* ± *m*) [28] were used to mathematically process the difference in the homology of the studied strains Chlamydia trachomatis (NPL, PL, PL2, and PL3) and Chlamydophila pneumoniae (PN, PN2, and B) with the strains of chlamydia found in GenBank NCBI. All mathematical processing was done in Microsoft Excel 2007. 

## 3. Results and Discussion

Our previous studies established that the strain Chlamydia trachomatis-NPL obtained from monkeys does not have any plasmids. This strain is less virulent than the plasmid-carrying strains Chlamydia trachomatis-PL and Chlamydia trachomatis-PL2, isolated from humans and the reference strain Chlamydia trachomatis-Burkhan (Chlamydia trachomatis-PL3) [16, 17]. Plasmids contain a gene that codes for the membrane protein pgp3 in chlamydia; this protein plays a role in the immune response of the organism to pathogens by inducing the generation of inflammatory cytokines in the macrophages and activating an inflammatory reaction [29]. Plasmids also play a dominant role in the accumulation of glycogen in chlamydia inclusions, which is explained by the presence in plasmids of sequences specific chromosome genes (pgi, mrsA1, glgA, glgB, glgX, and glgP) responsible for the metabolism of glycogen and performing the function of their transcription regulator. The Chlamydia trachomatis strains that do not carry the critical plasmid cause asymptomatic development of the disease (one was obtained from a patient suffering from proctocolitis and characterised as an L2 serotype L2 [30], another was taken from a patient suffering from asymptomatic urethritis and characterised as serovar B [31], a third clinical isolate, C599 was taken from the urethra of a patient with an asymptomatic condition and after being sequenced was characterised as serovar E/Bour [32]). 50% of the time an infecting dosage of a strain not carrying a plasmid is 400 times the dose of a plasmid-carrying strain [33]. While the majority of the Chlamydia trachomatis strains obtained from humans carry plasmids, 54% of the strains obtained from monkeys do not have plasmids. This explains the fact that monkeys suffering from chlamydia-caused conditions present with less clear symptoms than humans [17]. 

The cultivation of Chlamydia trachomatis strains in cells is accompanied either by the formation of multiple intracellular chlamydia inclusions or by the emergence of one big inclusion. This is first of all related to the expression of the *incA* gene at the level of the IncA protein (family of Inc-proteins—IncA, IncB, IncC, IncE, and IncG), which is part of the composition of the membrane inclusions and is responsible for vacuolisation. If there is no expression of this gene at all, the chlamydia inclusions multiply [34, 35]. The IncA protein has a function in the formation of secondary inclusions that allow chlamydia to form intracellular recesses where they can grow and continuously infect the cells forming after the division of the mother cell [36]. When the plasmid-free strain Chlamydia trachomatis-NPL—genotype E cultivates multiple intracellular chlamydia inclusions (vacuoles) are observed inside the cell while in the case of the plasmid-carrying strains Chlamydia trachomatis-PL (obtained from monkeys) and Chlamydia trachomatis-PL2 (obtained from humans), which are G genotype, one big intracellular chlamydia inclusion forms [16].

Genotyping the Chlamydia trachomatis strains established that the strains obtained from monkeys were primarily genotypes E (42.3%) and G (57.7%); the plasmid carrying strain Chlamydia trachomatis-PL has genotype G; the plasmid-free strain Chlamydia trachomatis-NPL is genotype E. Strains of five serotypes were obtained from humans (K: 46.2%, G: 23.1%, E: 19.2%, F: 7.7%, J: 3.8%); the plasmid carrying strain Chlamydia trachomatis-PL2 manifests as genotype G [9, 11, 37].

**Figure 1:**
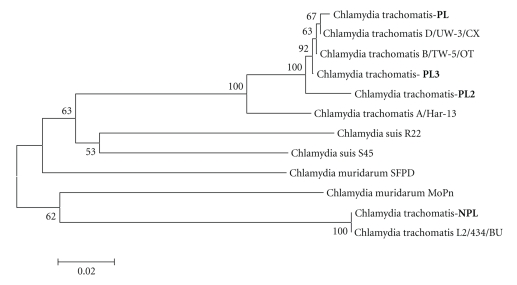
Dendrogram resulted from phylogenetic analysis of middle fragment of 16S–23S rRNA genes and 23S rRNA area of Chlamydia strains I segment by means of Neighbour-Joining method with execution of Bootstrap Test of Phylogeny (1000 repetitions), *p*-distance model demonstrating position of Chlamydia trachomatis strains being under investigation (PL: plasmid strain released from monkeys, PL2: plasmid strain released from humans, and PL3: reference strain and NPL: nonplasmid strain released from monkeys).

Comparative analysis of the homology was carried out and the evolutionary difference in the known sequences 15S-23S of the middle ribosome section and domain I of the 23 rRNA gene of different types of chlamydia was estimated, with comparisons being made with the strains being studied (Tables 1 and 2). The nucleotide sequences of the sequenced fragment of the plasmid-free strain Chlamydia trachomatis-NPL have a 99% homology and an evolutionary difference between the sequences of 0.672 to 0.690 (*s* = 0.019) compared with the strains Chlamydia trachomatis (A/Har-13, B/TW-5/OT, D/UW-3/CX), but it exhibits the greatest homology with Chlamydia trachomatis L2/434/BU (100%). In this case the difference between the sequences is 0 (*s* = 0), which means that the sequences of the strains NPL and L2/434/BU are the same despite the fact that NPL has no plasmid. This confirms the previously established similarity between strains that have no plasmid and those that have it [33]. With regard to other species of chlamydia the difference in homology exhibited by NPL was 15.92 ± 7.04%, the difference in the sequences is 0.690 to 0.755 (*s* = 0.018–0.019). The plasmid-carrying strains Chlamydia trachomatis-PL obtained from monkeys and Chlamydia trachomatis-PL2 obtained from humans are 98%–99% homologous with Chlamydia trachomatis (plasmid carrying strains), with the difference in the sequences being 0.016 to 0.697 for the strain PL (*s* = 0.005–0.019) and from 0.034 to 0.698 for PL2 (*s* = 0.008–0.019), with the homology differences from other types of chlamydia ranging from 13.31 ± 5.49% to 13.77 ± 5.97%, respectively. The nucleotide sequence of Chlamydia trachomatis—PL3 differs from that of Chlamydia trachomatis (A/Har-13, B/TW-5/OT, D/UW-3/CX, L2/434/BU) by 1%–2%, with the difference between the sequences ranging from 0.002 to 0.690 (*s* = 0.002–0.019). The difference in the homology of this strain and that of other species of chlamydia of both the Chlamydia and Chlamydophila genus is 13.15 ± 5.75%, with genetic distances between the sequences ranging from 0.426 to 0.691 (*s* = 0.019–0.021). When the evolutionary difference in the nucleotide sequences of the studied strains Chlamydia trachomatis between each other was studied, the following results were obtained: PL/PL2-0.019 (*s* = 0.006), PL/PL3: 0.052 (*s* = 0.009), PL/NPL: 0.697 (*s* = 0.019), PL2/PL3: 0.033 (*s* = 0.007), NPL/PL3: 0.690 (*s* = 0.019), and NPL/PL2: 0.698 (*s* = 0.019). Consequently the plasmid-carrying monkey strain Chlamydia trachomatis-PL is the closest to the PL2 strain obtained from humans, while the plasmid-free strain NPL is closer to the reference NPL strain, but nevertheless this still could not be put in different clusters when the phylogenetic tree found in Figures 1 and 3 was constructed. Additionally, we also estimated the nucleotide sequence 16S–23S of the middle ribosome section and domain I of the 23S rRNA gene of the studied plasmid-carrying and plasmid-free strains Chlamydia trachomatis to get a better estimate of the aforementioned evolutionary difference in the nucleotide sequences between these strains (Figures 4–6). When comparing the monkey strains Chlamydia trachomatis-PL-genotype G and Chlamydia trachomatis-NPL-genotype E, a difference was identified inside a section of 483 nucleotide pairs in length (Figure 4). A similar tendency was found when Chlamydia trachomatis-PL2-genotype G (human) was compared with Chlamydia trachomatis-NPL-genotype E (monkey), where differences can be observed in a section of 197 nucleotide pairs in length (Figure 5). When Chlamydia trachomatis-PL2 (human) was compared with Chlamydia trachomatis-PL (monkey), isolated differences were found (within a section 462 nucleotide pairs in length); both strains are genotype G (Figure 6).

The studied strains of the Chlamydophila pneumoniae species (PN, PN2 and B) exhibit a 98% and 99% homology with Chlamydophila pneumoniae N16 and Chlamydophila pneumoniae TW-183, respectively. The evolutionary differences between the sequences of the strains PN2, B, TW-183, and N16 are between 0.066 and 0.128 (*s* = 0.010–0.014); between the monkey strain PN and TW-183 it makes 0.002 (*s* = 0.002) with a homology of 99%; between PN and N16 it makes 0.190 (*s* = 0.016) with a homology of 98%. Thus PN2, B, and PN are close to Chlamydophila pneumoniae TW-183 in terms of homology and evolutionary differences between their nucleotide sequences. The difference in homology between PN2, B, PN, and other species is as follows: PN2: 6.0 ± 2.75%; B: 13.66 ± 3.73%; and PN: 13.73 ± 3.78%; the evolutionary difference between the nucleotide sequences varies from 0.545 to 0.719 (*s* = 0.019–0.021). Estimating the evolutionary difference in the nucleotide sequences between the studied strains of Chlamydophila pneumonia (PN2: human strain, PN: monkey strain, B: reference strain), we established the following: PN2/PN: 0.067 (*s* = 0.010); PN2/B: 0.0 (*s* = 0); and PN/B: 0.067 (*s* = 0.010). PN2 and the reference strain B are the closest to each other and are at the same evolutionary level; however, the insignificance of the difference from PN means that all of the three studied strains of Chlamydophila pneumonia (human and monkey) are very similar to each other. 

The homology between strain Chlamydophila pneumonia: PN, isolated from monkeys, and human strains of Chlamydophila pneumoniae amounted to 98%–99%; plasmid-free strain Chlamydia trachomatis: NPL was completely identical to Chlamydia trachomatis L2/434/BU (homology 100%), plasmid-carrying strain Chlamydia trachomatis: PL both with human and with known Chlamydia trachomatis strains from GenBank NCBI had 98%–99% homology, human Chlamydophila pneumoniae strains (PN2 and B) and Chlamydophila pneumoniae TW-183%: 99%; strains Chlamydia trachomatis (PL2, PL3) were homologous to Chlamydia trachomatis representatives (98%–100%).

In order to examine the relationship between plasmid-free Chlamydia trachomatis-NPL and strains of Chlamydia genus phylogenetically, the dendrogram was designed (p. 1), it shows that the strain is a representative of Chlamydia trachomatis species and is included into one cluster with Chlamydia trachomatis L2/434/BU.

The phylogenetic tree of examined plasmid-carrying Chlamydia trachomatis strains isolated from humans and monkeys (PL2 and PL), including reference strain Chlamydia trachomatis-Burkhun (PL3), with strains from Chlamydia genus is presented on the Figure 1. Strains PL, PL2, and PL3 are united into one combined cluster with Chlamydia trachomatis (B/TW-5/OT, D/UW-3/CX, A/Har-13), but into separate clusters with L2/434/BU.

The phylogenetic tree of Chlamydophila pneumoniae strains (PN, PN2, and B) with strains from Chlamydia genus (p. 2) reflects the relationship between strains PN isolated from monkeys, with strain, isolated from humans (PN2), reference strain Chlamydophila pneumoniae-B and strains Chlamydophila pneumoniae TW-183, Chlamydophila pneumoniae N16. Strain PN is included into one subcluster with Chlamydophila pneumoniae TW-183, this fact suggests that the strain is a member of Chlamydophila pneumoniae species, in one cluster with Chlamydophila pneumoniae-PN2 and Chlamydophila pneumoniae-B, which are in one subcluster according to evolutional discrepancy. 

Phylogenetic analysis of strains Chlamydophila pneumoniae and Chlamydia trachomatis, isolated from monkeys, showed that these strains are really the members of Chlamydophila genus and Chlamydia genus according to general location in clusters on dendrograms with analogical human strains.

Considering the phylogenetic aspect of the relation between the plasmid-free strain Chlamydia trachomatis-NPL with strains of the Chlamydia genus, we constructed a tree diagram (Figure 1), which indicates that this strain is a Chlamydia trachomatis and is in the same cluster as Chlamydia trachomatis L2/434/BU.

The phylogenetic tree of the studied plasmid-carrying strains of Chlamydia trachomatis, obtained from humans and monkeys (PL2 and PL), including the reference strain of Chlamydia trachomatis-Burkhan (PL3), with the strain of Chlamydia is presented in Figure 1. Strains PL, PL2, and PL3 are grouped into the same cluster with Chlamydia trachomatis (B/TW-5/OT, D/UW-3/CX, A/Har-13), but they are placed in a different cluster than L2/434/BU.

**Figure 2:**
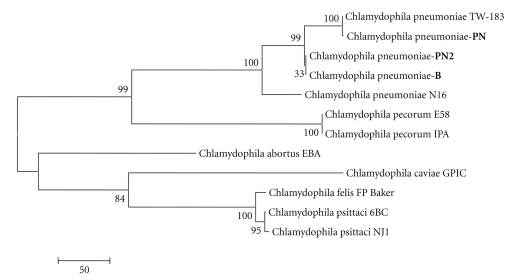
Dendrogram resulted from phylogenetic analysis of middle fragment of 16S–23S rRNA genes and 23S rRNA area of Chlamydophila Chlamydia strains I segment by means of Maximum Parsimony method with execution of Bootstrap Test of Phylogeny (1000 repetitions), *p*-distance model reflecting position of Chlamydophila pneumoniae strains being under investigation (PN: monkey strain, PN2: human strain, and B: reference strain).

**Figure 3:**
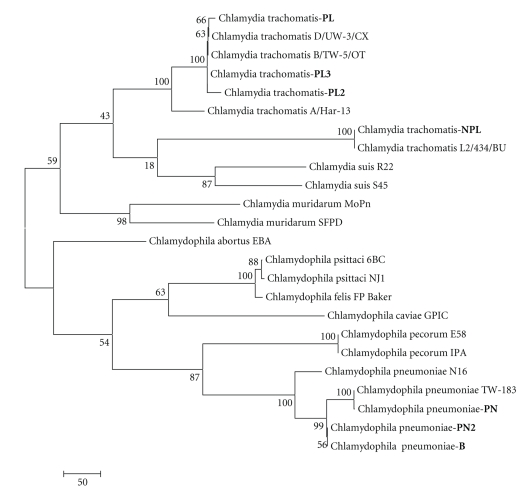
Dendrogram resulted from phylogenetic analysis of middle fragment of 16S–23S rRNA genes and 23S rRNA area of Chlamydia strains I segment by means of Maximum Parsimony method with execution of Bootstrap Test of Phylogeny (1000 repetitions). Strains being under investigation: Chlamydophila pneumoniae (PN: monkey strain, PN2: human strain, and B: reference strain) and Chlamydia trachomatis (PL: plasmid strain released from monkeys, PL2: plasmid strain released from humans, and PL3: reference strain and NPL-nonplasmid strain released from monkeys).

**Figure 4:**
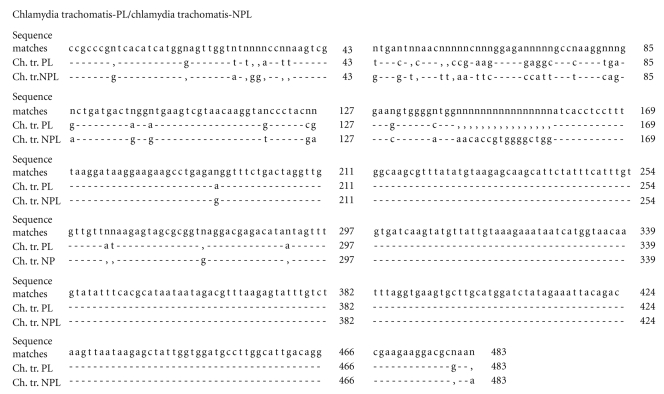
Comparative analysis of 16S–23S nucleotide sequence clusters of the middle ribosomal cluster and 23S rRNA I domain of plasmid (PL) and nonplasmid (NPL) Chlamydia trachomatis strains released from monkeys. (-)—match of nucleotides in the sequence, (n)—mismatch of nucleotides in the sequence, (,)—absence of nucleotides in the sequence, (43-483)—nucleotide sequence size.

**Figure 5:**
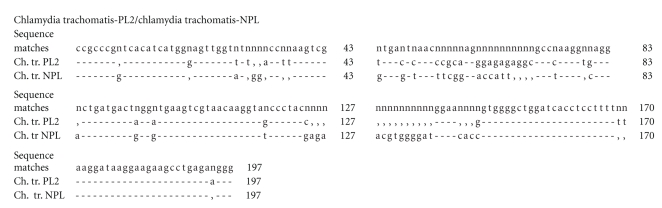
Comparative analysis of 16S–23S nucleotide sequence clusters of the middle ribosomal cluster and 23S rRNA I domain of nonplasmid (NPL) Chlamydia trachomatis strains released from monkeys and plasmid (PL2) Chlamydia trachomatis strains released from humans. (-)—match of nucleotides in the sequence, (n)—mismatch of nucleotides in the sequence, (,)—absence of nucleotides in the sequence, (43-197)—nucleotide sequence size.

**Figure 6:**
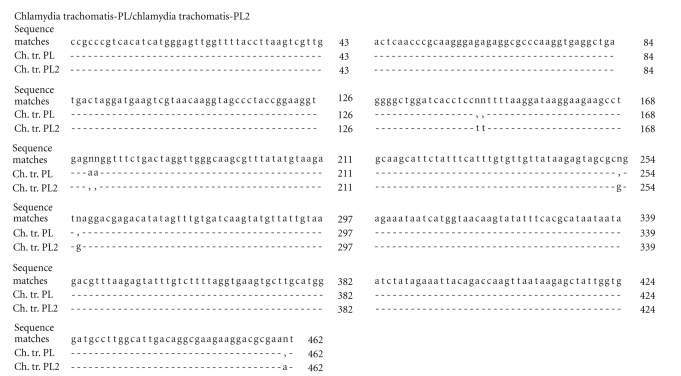
Comparative analysis of 16S–23S nucleotide sequence clusters of the middle ribosomal cluster and 23S rRNA I domain of plasmid Chlamydia trachomatis strains released from monkeys (PL strain) and from humans (PL2 strain). (-)—match of nucleotides in the sequence, (n)—mismatch of nucleotides in the sequence, (,)—absence of nucleotides in the sequence, (43-462)—nucleotide sequence size.

The phylogenetic tree of the strains of Chlamydophila pneumoniae (PN, PN2, and B) with strains of the Chlamydophila genus (Figure 2) reflects the relation between the PN strain obtained from monkeys with the PN2 strain obtained from humans, the reference strain of Chlamydophila pneumoniae-B and the strains of Chlamydophila pneumoniae TW-183, and Chlamydophila pneumoniae N16. The PN strain was place in the same subcluster with Chlamydophila pneumoniae TW-183. It is also in the same cluster with Chlamydophila pneumoniae-PN2 and Chlamydophila pneumoniae-B, which are in the same subcluster in accordance with their small evolutionary differences. 

Thus phylogenetic analysis of strains of Chlamydia trachomatis and Chlamydophila pneumoniae obtained from monkeys and humans has allowed us to establish their place in the phylogenetic tree of the Chlamydiaceae family. It has been established that there is a close evolutionary relation between the studied original species of Chlamydia and Chlamydophila and similar species about which there are records in GenBank. For the first time, it has been demonstrated that there are differences in the nucleotide sequence 16S–23S of the middle ribosome section and domain I of the 23S rRNA gene of plasmid-carrying and plasmid-free strains of Chlamydia trachomatis obtained from monkeys and humans, if they have different genotypes (group B- B, Ba, D, Da, E, L1, L2, L2a; intermediary group- F, G, Ga). Malfunction of the expression of the chromosome gene incA that leads to a breakdown in the development and life cycle of chlamydia and their virulence may also be linked to possible changes in the nucleotide sequence of this gene. 

## Figures and Tables

**Table 1:** Comparative analysis results concerning homology of the nucleotide sequence 16S–23S of the middle ribosomal cluster and 23S rRNA I gene domain of the Chlamydia strains being investigated with analogous sequences of other strains.

Chlamydia strains from the GenBank database	Homology percentage of the strains being investigated related to known Chlamydia strains from the GenBank database													
	Chlamydia trachomatis-NPL		Chlamydia trachomatis-PL		Chlamydophila pneumoniae PN		Chlamydia trachomatis-PL2		Chlamydophila pneumoniae-PN2		Chlamydophila pneumoniae-B		Chlamydia trachomatis-PL3	
	hm$\hat{\;}$	d**	hm$\hat{\;}$	d**	hm$\hat{\;}$	d**	hm$\hat{\;}$	d**	hm$\hat{\;}$	d**	hm$\hat{\;}$	d**	hm$\hat{\;}$	d**
Chl. abortus EBA	78	22	83	17	90	10	83	17	95	5	90	10	83	17
Chl. psittaci 6BC	78	22	84	16	90	10	84	16	94	6	90	10	84	16
Chl. psittaci NJ1	78	22	84	16	91	9	83	17	96	4	91	9	84	16
Chl. caviae GPIC	79	21	84	16	90	10	84	16	95	5	90	10	85	15
Chl. felis FP Baker	79	21	85	15	90	10	85	15	85	15	90	10	85	15
Chl. pneumoniae N16	82	18	79	21	98	2	82	18	98	2	98	2	82	18
Chl. pneumoniae TW-183	83	17	80	20	99	1	84	16	99	1	99	1	82	18
Chl. pecorum E58	80	20	82	18	90	10	82	18	97	3	90	10	82	18
Chl. pecorum IPA	80	20	82	18	90	10	82	18	97	3	90	10	82	18
Ch. trachomatis A/Har13	99	1	99	1	82	18	99	1	94	6	82	18	100	0
Ch. trachomatis B/TW-5/OT	99	1	99	1	82	18	99	1	94	6	82	18	99	1
Ch. trachomatis D/UW-3/CX	99	1	99	1	83	17	99	1	94	6	83	17	99	1
Ch. trachomatis L2/434/BU	100	0	98	2	83	17	98	2	94	6	83	17	98	2
Ch. suis R22	94	6	95	5	84	16	95	5	94	6	84	16	95	5
Ch.suis S45	94	6	95	5	83	17	95	5	93	7	84	16	95	5
Chl. muridarum MoPn	94	6	94	6	83	17	94	6	94	6	83	17	95	5
Chl. muridarum SFPD	94	6	94	6	83	17	94	6	94	6	83	17	95	5
difference in homology (*M *±*m*)*	15.92 ± 7.04		13.77 ± 5.97		13.73 ± 3.78		13.31 ± 5.49		6.0 ± 2.75		13.66 ± 3.73		13.15 ± 5.75	

Notes: homology grade of sequences 16S–23S of the middle ribosomal cluster and 23S rRNA I gene domain of Chlamydia trachomatis (NPL, PL, PL2, and PL3) and Chlamydophila pneumoniae (PN, PN2 and B) strains being investigated and with various Chlamydia types as published in GenBank NCBI, executed by BLAST program (http://blast.ncbi.nlm.nih.gov/Blast.cgi/); hm$\hat{\;}$ - strains homology percentage; d**: percentage of difference in strains homology; *: difference in homology between the strains being investigated and other Chlamydia types (*M *±* m*).

**Table 2:** Estimation of evolutional difference between nucleotide sequences and standard error of estimation (*s*) of middle fragment of16S–23S rRNA genes and 23S rRNA area of Chlamydia spp. I segment.

No.	strain	PL3	PL	PL2	NPL	MoPn	SFPD	E58	R22	L2/434/BU	S45	A/Ha-13	B/TW-5/OT	D/UW3/CX	EBA	GPIC	IPA	N16	TW-183	6BC	PN2	NJ1	B	PN	FPBa ker
1	PL3		0.009	0.007	0.019	0.020	0.021	0.019	0.021	0.019	0.021	0.015	0.002	0.002	0.021	0.019	0.019	0.019	0.019	0.021	0.020	0.021	0.020	0.019	0.021
2	PL	0.052		0.006	0.019	0.020	0.021	0.019	0.021	0.019	0.021	0.016	0.005	0.005	0.021	0.019	0.019	0.020	0.020	0.021	0.020	0.021	0.020	0.020	0.021
3	PL2	0.033	0.019		0.019	0.021	0.021	0.019	0.021	0.019	0.021	0.016	0.008	0.008	0.021	0.019	0.019	0.019	0.019	0.021	0.019	0.021	0.019	0.019	0.021
4	NPL	0.690	0.697	0.698		0.019	0.019	0.018	0.019	0.000	0.019	0.019	0.019	0.019	0.019	0.018	0.018	0.019	0.019	0.018	0.019	0.018	0.019	0.019	0.018
5	MoPn	0.579	0.583	0.578	0.709		0.021	0.019	0.020	0.019	0.020	0.021	0.020	0.021	0.020	0.019	0.019	0.020	0.020	0.020	0.020	0.020	0.020	0.020	0.020
6	SFPD	0.534	0.545	0.534	0.719	0.440		0.020	0.021	0.019	0.021	0.021	0.021	0.021	0.021	0.020	0.020	0.019	0.019	0.021	0.019	0.021	0.019	0.019	0.021
7	E58	0.691	0.691	0.676	0.743	0.674	0.664		0.019	0.018	0.020	0.019	0.019	0.019	0.020	0.020	0.000	0.021	0.021	0.019	0.021	0.019	0.021	0.021	0.019
8	R22	0.459	0.462	0.491	0.690	0.652	0.486	0.698		0.019	0.020	0.021	0.021	0.021	0.021	0.019	0.019	0.020	0.020	0.021	0.020	0.021	0.020	0.020	0.021
9	L2/434/BU	0.690	0.697	0.698	0.000	0.709	0.719	0.743	0.690		0.019	0.019	0.019	0.019	0.019	0.018	0.018	0.019	0.019	0.018	0.019	0.018	0.019	0.019	0.018
10	S45	0.426	0.429	0.459	0.709	0.638	0.512	0.671	0.391	0.709		0.021	0.021	0.021	0.021	0.019	0.020	0.019	0.019	0.021	0.019	0.021	0.019	0.019	0.021
11	A/Ha-13	0.153	0.172	0.186	0.672	0.550	0.555	0.697	0.445	0.672	0.422		0.015	0.015	0.021	0.020	0.019	0.019	0.019	0.021	0.019	0.021	0.019	0.019	0.021
12	B/TW-5/OT	0.002	0.017	0.034	0.691	0.579	0.534	0.691	0.460	0.691	0.428	0.155		0.002	0.021	0.019	0.019	0.019	0.019	0.021	0.020	0.021	0.020	0.019	0.021
13	D/UW3/CX	0.003	0.016	0.036	0.690	0.578	0.536	0.691	0.459	0.690	0.426	0.157	0.002		0.021	0.019	0.019	0.019	0.019	0.021	0.020	0.021	0.020	0.019	0.021
14	EBA	0.491	0.488	0.503	0.709	0.653	0.540	0.660	0.505	0.709	0.531	0.516	0.490	0.491		0.020	0.020	0.020	0.020	0.021	0.020	0.021	0.020	0.020	0.021
15	GPIC	0.691	0.690	0.700	0.755	0.681	0.648	0.653	0.684	0.755	0.674	0.664	0.690	0.688	0.648		0.020	0.019	0.019	0.021	0.019	0.021	0.019	0.019	0.021
16	IPA	0.691	0.691	0.676	0.743	0.674	0.664	0.000	0.698	0.743	0.671	0.697	0.691	0.691	0.660	0.653		0.021	0.021	0.019	0.021	0.019	0.021	0.021	0.019
17	N16	0.674	0.671	0.676	0.707	0.660	0.707	0.550	0.671	0.707	0.678	0.679	0.674	0.674	0.624	0.703	0.550		0.016	0.021	0.014	0.021	0.014	0.016	0.021
18	TW-183	0.672	0.669	0.674	0.719	0.650	0.690	0.545	0.667	0.719	0.690	0.678	0.672	0.672	0.621	0.693	0.545	0.191		0.020	0.010	0.020	0.010	0.002	0.020
19	6BC	0.526	0.529	0.538	0.734	0.662	0.557	0.688	0.512	0.734	0.526	0.514	0.526	0.528	0.503	0.560	0.688	0.481	0.638		0.021	0.004	0.021	0.020	0.007
20	PN2	0.671	0.667	0.672	0.716	0.652	0.691	0.545	0.666	0.716	0.681	0.676	0.671	0.671	0.617	0.700	0.545	0.128	0.066	0.574		0.021	0.000	0.010	0.021
21	NJ1	0.528	0.531	0.540	0.734	0.664	0.553	0.688	0.512	0.734	0.526	0.517	0.528	0.529	0.507	0.562	0.688	0.474	0.633	0.009	0.569		0.021	0.020	0.008
22	B	0.671	0.667	0.672	0.716	0.652	0.691	0.545	0.666	0.716	0.681	0.676	0.671	0.671	0.617	0.700	0.545	0.128	0.066	0.574	0.000	0.569		0.010	0.021
23	PN	0.674	0.671	0.676	0.719	0.652	0.691	0.547	0.669	0.719	0.691	0.679	0.674	0.674	0.622	0.693	0.547	0.190	0.002	0.640	0.067	0.634	0.067		0.020
24	FPBaker	0.522	0.528	0.538	0.733	0.666	0.550	0.700	0.507	0.733	0.526	0.514	0.522	0.524	0.509	0.564	0.700	0.479	0.638	0.031	0.576	0.036	0.576	0.640	

Notes: The obtained results are based on 24 strains sequences pairwise analysis. Calculations were made with MEGA 4.1 program by means of *p*-distance method [27]. Numbers below the diagonal show genetic distances between cluster sequences. The standard error of evolutional difference estimation (*s*) is calculated with MEGA 4.1 program by means of analytic formulae and is shown above the diagonal.

## References

[1] Everett KDE, Bush RM, Andersen AA (1999). Emended description of the order Chlamydiales, proposal of parachlamydiaceae fam. nov. and Simkaniaceae fam. nov., each containing one monotypic genus, revised taxonomy of the family Chlamydiaceae, including a new genus and five new species, and standards for the identification of organisms. *International Journal of Systematic Bacteriology*.

[2] Fukushi H, Hirai K (1992). Proposal of Chlamydia pecorum sp. nov. for *Chlamydia* strains derived from ruminants. *International Journal of Systematic Bacteriology*.

[3] Hartley JC, Kaye S, Stevenson S, Bennett J, Ridgway G (2001). PCR detection and molecular identification of Chlamydiaceae species. *Journal of Clinical Microbiology*.

[4] Everett KDE, Andersen AA (1997). The ribosomal intergenic spacer and domain I of the 23S rRNA gene are phylogenetic markers for *Chlamydia* spp. *International Journal of Systematic Bacteriology*.

[5] Grayston JT, Kuo C-C, Campbell LA, Wang S-P (1989). *Chlamydia pneumoniae* sp. nov. for *Chlamydia* sp. strain TWAR. *International Journal of Systematic Bacteriology*.

[6] Amann R, Springer N, Schönhuber W (1997). Obligate intracellular bacterial parasites of acanthamoebae related to *Chlamydia* spp. *Applied and Environmental Microbiology*.

[7] Birtles RJ, Rowbotham TJ, Storey C, Marrie TJ, Raoult D (1997). *Chlamydia*-like obligate parasite of free-living amoebae. *Lancet*.

[8] Kahane S, Everett KDE, Kimmel N, Friedman MG (1999). Simkania negevensis strain Z(T): growth, antigenic and genome characteristics. *International Journal of Systematic Bacteriology*.

[9] Kong F, Gilbert GL (2004). Postgenomic taxonomy of human ureaplasmas—a case study based on multiple gene sequences. *International Journal of Systematic and Evolutionary Microbiology*.

[10] Liu K, Knabel SJ, Dudley EG (2009). rhs genes are potential markers for multilocus sequence typing of Escherichia coli O157:H7 strains. *Applied and Environmental Microbiology*.

[11] Matsumoto T, Kawakubo M, Shiohara M (2009). Phylogeny of a novel “helicobacter heilmannii” organism from a Japanese patient with chronic gastritis based on DNA sequence analysis of 16S rRNA and urease genes. *Journal of Microbiology*.

[12] Pudjiatmoko, Fukushi H, Ochiai Y, Yamaguchi T, Hirai K (1997). Phylogenetic analysis of the genus *Chlamydia* based on 16S rRNA gene sequences. *International Journal of Systematic Bacteriology*.

[13] Rurangirwa FR, Dilbeck PM, Crawford TB, McGuire TC, McElwain TF (1999). Analysis of the 16S rRNA gene of microorganism WSU 86–1044 from an aborted bovine foetus reveals that it is a member of the order Chlamydiales: proposal of Waddliaceae fam. nov., waddlia chondrophila gen. nov., sp. nov. *International Journal of Systematic Bacteriology*.

[14] Takahashi M, Yukphan P, Yamada Y, Suzuki K-I, Sakane T, Nakagawa Y (2006). Intrageneric structure of the genus Gluconobacter analyzed by the 16S rRNA gene and 16S-23S rRNA gene internal transcribed spacer sequences. *Journal of General and Applied Microbiology*.

[15] Gaydos CA, Palmer L, Quinn TC, Falkow S, Eiden JJ (1993). Phylogenetic relationship of *Chlamydia pneumoniae* to Chlamydia psittaci and *Chlamydia trachomatis* as determined by analysis of 16S ribosomal DNA sequences. *International Journal of Systematic Bacteriology*.

[16] Grechishnikova OG, Slobodenyuk VV, Aleshkin VA (2009). Phenotypic charac-teristics of *Chlamydia* strains released from humans and monkeys by means of culture technique. *Immunopathology, Allergology, Infectology*.

[17] Slobodenyuk VV, Aleshkin VA, Lapin BA (2009). PCR detection and identification of *Chlamydia* germs in humans and monkeys. *Immunopathology, Allergology, Infectology*.

[18] Tamura K, Dudley J, Nei M, Kumar S (2007). MEGA4: molecular evolutionary genetics analysis (MEGA) software version 4.0. *Molecular Biology and Evolution*.

[19] Pickett MA, Everson JS, Pead PJ, Clarke IN (2005). The plasmids of *Chlamydia trachomatis* and Chlamydophila pneumoniae (N16): accurate determination of copy number and the paradoxical effect of plasmid-curing agents. *Microbiology*.

[20] Thomas NS, Lusher M, Storey CC, Clarke IN (1997). Plasmid diversity in *Chlamydia*. *Microbiology*.

[21] Sriprakash KS, Macavoy ES (1987). Characterization and sequence of a plasmid from the trachoma biovar of *Chlamydia trachomatis*. *Plasmid*.

[22] Comanducci M, Ricci S, Cevenini R, Ratti G (1990). Diversity of the *Chlamydia trachomatis* common plasmid in biovars with different pathogenicity. *Plasmid*.

[23] Azuma Y, Hirakawa H, Yamashita A (2006). Genome sequence of the cat pathogen, Chlamydophila felis. *DNA Research*.

[24] Read TD, Myers GSA, Brunham RC (2003). Genome sequence of Chlamydophila caviae (Chlamydia psittaci GPIC): examining the role of niche-specific genes in the evolution of the Chlamydiaceae. *Nucleic Acids Research*.

[25] Carlson JH, Porcella SF, McClarty G, Caldwell HD (2005). Comparative genomic analysis of *Chlamydia trachomatis* oculotropic and genitotropic strains. *Infection and Immunity*.

[26] Thomson NR, Holden MTG, Carder C (2008). *Chlamydia trachomatis*: genome sequence analysis of lymphogranuloma venereum isolates. *Genome Research*.

[27] Read TD, Brunham RC, Shen C (2000). Genome sequences of *Chlamydia trachomatis* MoPn and *Chlamydia pneumoniae* AR39. *Nucleic Acids Research*.

[28] Yunkerov VI, Grigor’ev SG (2005). *Mathematical and Statistical Analysis of Medical Investigation Data*.

[29] Li Z, Chen D, Zhong Y, Wang S, Zhong G (2008). The Chlamydial plasmid-encoded protein pgp3 is secreted into the cytosol of Chlamydia-infected cells. *Infection and Immunity*.

[30] Peterson EM, Markoff BA, Schachter J, de la Maza LM (1990). The 7.5-kb plasmid present in *Chlamydia trachomatis* is not essential for the growth of this microorganism. *Plasmid*.

[31] Farencena A, Comanducci M, Donati M, Ratti G, Cevenini R (1997). Characterization of a new isolate of *Chlamydia trachomatis* which lacks the common plasmid and has properties of biovar trachoma. *Infection and Immunity*.

[32] Stothard DR, Williams JA, van der Pol B, Jones RB (1998). Identification of a *Chlamydia trachomatis* serovar e urogenital isolate which lacks the cryptic plasmid. *Infection and Immunity*.

[33] Carlson JH, Whitmire WM, Crane DD (2008). The *Chlamydia trachomatis* plasmid is a transcriptional regulator of chromosomal genes and a virulence factor. *Infection and Immunity*.

[34] Pannekoek Y, Spaargaren J, Langerak AAJ, Merks J, Morré SA, van der Ende A (2005). Interrelationship between polymorphisms of *IncA*, fusogenic properties of *Chlamydia trachomatis* strains, and clinical manifestations in patients in The Netherlands. *Journal of Clinical Microbiology*.

[35] Suchland RJ, Rockey DD, Bannantine JP, Stamm WE (2000). Isolates of *Chlamydia trachomatis* that occupy nonfusogenic inclusions lack *IncA*, a protein localized to the inclusion membrane. *Infection and Immunity*.

[36] Suchland RJ, Rockey DD, Weeks SK, Alzhanov DT, Stamm WE (2005). Development of secondary inclusions in cells infected by *Chlamydia trachomatis*. *Infection and Immunity*.

[37] Slobodenyuk VV, Karaulov AV, Aleshkin VA (2009). Genetic typing and analysis of antibiotic resistance of *Chlamydia trachomatis* strains released from humans and monkeys. *Immunopathology, Allergology, Infectology*.

